# Foreign Items

**Published:** 1869-10

**Authors:** 


### Foreign Items.

BARON LARRY, in his important work on Trephining, in traumatic lesions of the skull (published by Victor Masson & Co., Paris), thus summarizes the indications and contra-indications for this operation.

*Indications.*—Trephining should be practiced in traumatic lesions of the head, if symptoms, clearly localized and circumscribed, persist, and if all other means are unavailing, in the following cases:

1st. In fractures of the vault, by perforation or with imbedding of the fragments, whenever the injury to deep-seated parts occasions grave and continuous consequences, whilst remedial measures other than trephining may be impossible or inefficacious.

2nd. In fractures complicated with the perforation of foreign bodies of the thickness of the cranium, or with penetration into the superficial layers of the brain with persistence of symptomatic accidents, if the extraction of the foreign body can be effected in no other way.

3rd. In the different mechanical lesions of the head, complicated with grave and persistent cerebral symptoms, such as contusion and compression of the brain, or even prolonged hemiplegia with extravasation of blood or of pus, considered as circumscribed, provided that the lesion can be localized, and provided, especially, that active therapeutics remain insufficient.

*Contra-Indications.*—Trephining should not be attempted in cases of lesion speedily or necessarily fatal, nor in those presumed to be curable by other means.

1st. When a foreign substance, lost in the deep portions of the brain, has passed beyond the reach of instruments.

2nd. If blood or pus, extravasated within the cranium, does not appear to form a focus in relation with the opening in the bone.

3rd. In every case of fracture not complicated with firm imbedding of the fragments, nor with prolonged phenomena of compression or paralysis.

4th. In the condition of cerebral commotion or coma, with or without localized lesions.

5th. In indeterminate or epileptiform convulsions, not persistent and susceptible of cure.

6th. In diffuse inflammation of the brain or its membranes, when clearly appreciable.—*Gazette des Hopitaux.*

On Thursday, August 19, M. Bailly, in the course of a clinical lecture upon a case of very profuse uterine hæmorrhage supervening in the seventh month of pregnancy, assigned for metrorrhagia in pregnant women, the following causes.

The first, and beyond doubt much the most frequent cause during the last months of pregnancy, is the "*vicious*" insertion of the placenta into the uterine neck.

The second, is the rupture of the utero-placental vessels (the insertion of the placenta being normal) under the influence of a congestive molimen.

The third source of this phenomenon the lecturer locates in the vessels of the mucous surface of the uterus, apart from the placenta, which ordinarily but slightly developed, may occasionally, under the menstrual impulse, give origin to a flow of blood.

These three sorts of hæmorrhage all originate within the body of the uterus. The remainder pertain to the neck, and of these, the first results from cancerous disease located in the neck, which becomes predisposed to hæmorrhage by reason of the access of blood to the uterus induced by pregnancy. These hæmorrhages occur most frequently during the latter period of pregnancy, for the reasons applicable to cases of placenta prævia.

But again, aside from any cancerous, syphilitic or other malignant disease, the mucous surface of the uterine neck, during pregnancy, sometimes becomes granular, and these granulations becoming fungous and very vascular, bleed at the slightest touch, and they are, moreover, peculiarly sensitive at the menstrual epoch. Several examples of this sort have even been known to occur at the first menstrual period subsequent to the removal of the ovaries of the body, and even of the supra-vaginal portion of the neck of the uterus. This form of hæmorrhage is frequently, although not invariably, indicative of pre-existing disease of the cervix.—*Gazette des Hopitaux.*

M. Good thus tabulates the statistics of 112 coxo-femoral resections performed subsequent to 1860:

Of the 112 cases, 52 (or 46.43 per cent.) were successful and 60 (or 53.57 per cent.) fatal. In relation to the nationalities, the cases are arranged as follows:

In Germany, 34 operations, 12 (35.29 per cent.) successful.

In England, 32 operations, 21 (95.62 per cent.) successful.

In America, 29 operations, 16 (55.17 per cent.) successful.

In France, 14 operations, and only 2 (those of MM. Sédillot & Backel, a Strasbourg) successful.

In Russia, 3 operations, 1 successful.

Of the 52 cures, 19 walked without support, and 9 with a simple cane. M. Good compares with these the results of the expectant treatment at the Hospital St. Eugenie (Paris), of 12 cases of suppurating coxalgia. One only resulted in spontaneous recovery, three remained stationary during several years, and eight died.—*Gazette des Hopitaux.*

M. Liebreich has communicated to the Academy of Sciences (Paris) three experiments made to test the anæsthetic and soporific properties of chloral. terchloride of aldheyde. The first, a hypodermic injection of one 1.57 gramme, in the case of an insane epileptic troubled with hallucinations and wakefulness. Five minutes afterwards the patient fell into a deep sleep, which continued four hours and a half. The two other experiments were made in the Charity Hospital, Berlin, under the direction of Professor Bardeleben. The first of these was followed by deep sleep, of several hours duration. The second was marked by decided anæsthesia, the particulars of which are detailed.

A case of metrorrhagia, of more than one month's duration, has been successfully treated, at the Necker Hospital, under the direction of M. Potain, by hot baths. There was no lesion of the uterus, and only a slight enlargement of the neck.

M. Bourdon, at la Charité, has succeeded in preventing the pitting of small-pox, by covering the face with cerate and sprinkling this thickly with starch, so as to form a paste.

Apropos to the discussion upon animal vaccination, M. Herard has made a comparative study of animal and Jennerian (human) vaccination, in four hundred and ninety-three new-born infants, and has been unable to detect any difference whatever between the effects of the two varieties of virus. They were identical.

